# Role of CD36 in cancer progression, stemness, and targeting

**DOI:** 10.3389/fcell.2022.1079076

**Published:** 2022-12-08

**Authors:** Sandra L. Guerrero-Rodríguez, Cecilia Mata-Cruz, Sonia M. Pérez-Tapia, Marco A. Velasco-Velázquez

**Affiliations:** ^1^ Pharmacology Department, School of Medicine, Universidad Nacional Autónoma de México, Mexico City, Mexico; ^2^ Graduate Program in Biochemical Sciences, Universidad Nacional Autónoma de México, Mexico City, Mexico; ^3^ Research and Development in Biotherapeutics Unit, National School of Biological Sciences, National Polytechnic Institute, Mexico City, Mexico; ^4^ National Laboratory for Specialized Services of Investigation Development and Innovation (I+D+i) for Pharma Chemicals and Biotechnological products LANSEIDI-FarBiotec-CONACyT, Mexico City, Mexico; ^5^ Immunology Department, National School of Biological Sciences, National Polytechnic Institute, Mexico City, Mexico

**Keywords:** CD36, oxLDL, cancer stem cells, metastasis, drug development

## Abstract

CD36 is highly expressed in diverse tumor types and its expression correlates with advanced stages, poor prognosis, and reduced survival. In cancer cells, CD36: 1) increases fatty acid uptake, reprogramming lipid metabolism; 2) favors cancer cell proliferation, and 3) promotes epithelial-mesenchymal transition. Furthermore, CD36 expression correlates with the expression of cancer stem cell markers and CD36^+^ cancer cells display increased stemness functional properties, including clonogenicity, chemo- and radioresistance, and metastasis-initiating capability, suggesting CD36 is a marker of the cancer stem cell population. Thus, CD36 has been pointed as a potential therapeutic target in cancer. At present, at least three different types of molecules have been developed for reducing CD36-mediated functions: blocking monoclonal antibodies, small-molecule inhibitors, and compounds that knock-down CD36 expression. Herein, we review the role of CD36 in cancer progression, its participation in stemness control, as well as the efficacy of reported CD36 inhibitors in cancer cell cultures and animal models. Overall, the evidence compiled points that CD36 is a valid target for the development of new anti-cancer therapies.

## 1 Introduction

CD36 is a membrane protein that belongs to the class B scavenger receptor family, which also includes scavenger B receptor type 1 (SCARB1) and lysosomal integral membrane protein 2 (LIMP-2) (Park, 2014). CD36 is physiologically expressed on the surface of multiple cells including adipocytes, monocytes, macrophages, platelets, endothelial cells, cardiomyocytes, dendritic cells, epithelial cells, erythrocytes, and muscle cells (Choromañska et al., 2017).

CD36 has 472 amino acids (aa) organized into: 1) two transmembrane domains; 2) an extracellular domain; and 3) two short cytoplasmic tails of 5–7 and 11–13 aa (NH2 and COOH terminal domains, respectively) (Park, 2014; Pepino et al., 2014; Hsieh et al., 2016). The crystallographic structure of CD36 ectodomain interacting with the CIDRα domain of PfEMP1 of *Plasmodium falciparum* has been published (Hsieh et al., 2016). CD36 has two entrances for long-chain fatty acids (FA) (Hsieh et al., 2016) and it has been proposed that the interaction site with FA ranges from aa 127 to 279 (Baillie et al., 1996). However, it is unclear the precise mechanism by which CD36 allows FA entrance into the cell. A recent hypothesis proposes that FA transmembrane gradient promotes their transport through CD36 cleft, allowing the FA to join the outer lipid layer for subsequent internalization by the “flip-flop” movement of the membrane (Glatz and Luiken, 2020; Jay et al., 2020). CD36 is also capable of interacting with oxidized low-density lipoproteins (oxLDL). Directed mutagenesis showed that aa 160 to 168 aa are essential for such function (Kar et al., 2008), overlapping the proposed FA binding site. The structural changes in CD36 during oxLDL transport are currently unknown. Finally, CD36 can bind other ligand proteins such as: 1) advanced glycation end products (Ohgami et al., 2002); 2) oxidized phospholipids (Gao et al., 2010); 3) amyloid proteins (El Khoury et al., 2003); and 4) thrombospondins (TSP) 1 and 2 (Klenotic et al., 2013).

CD36-activated signal transduction is mediated by the carboxy-terminal domain, which is able to interact with intracellular tyrosine kinases (Heit et al., 2013; Pepino et al., 2014; Yang et al., 2018; Hao et al., 2020). Due to its ability to interact with multiple ligands, CD36 participates in different functions: angiogenesis, cell adhesion, apoptosis, inflammatory processes, and lipid metabolism (Pepino et al., 2014; Zhao et al., 2018; Hao et al., 2020).

CD36 expression is regulated at both the transcriptional and post-translational levels, but the regulation differs between different cell types. Furthermore, CD36 function depends on the cellular context and its transcriptional and post-translational regulation. For example, under normal conditions, macrophages are responsible for the regulation of plasma lipoprotein metabolism through their different “scavenger” type receptors, including CD36. Increases in serum oxLDL or local cytokines (CSF, IL-4, and IL-10) levels stimulate CD36 expression in macrophages as a compensatory mechanism to return to the homeostatic state (Chen et al., 2022). CD36 is over-expressed and stimulate the accumulation of cholesterol, product of the degradation of oxLDL, within the macrophages, triggering their transformation into foam cells. In turn, foam cells are responsible for the development of initial lesions that lead to the formation of advanced atherosclerotic plaques (Chistiakov et al., 2017; Zingg et al., 2021).

**FIGURE 1:**
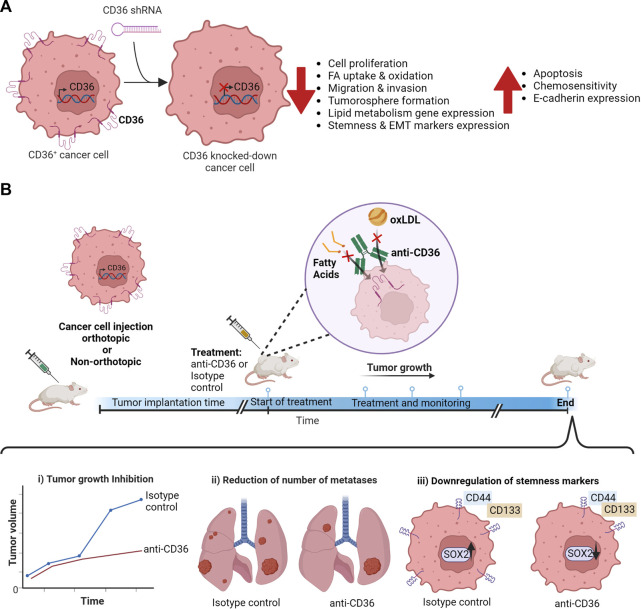
Impact of CD36 blockage on cancer cells. **(A)** Changes in cancer cell functions on CD36^+^ cancer cells generated by CD36 knock-down using CD36-targeting shRNA. **(B)** Experimental timeline summarizing the effects of anti-CD36 antibodies *in vivo.*

In cancer, CD36 expression promotes tumor development, metastasis, drug resistance (Table 1) and can modulate anti-cancer immunity (Pascual et al., 2017). Within tumors, CD36 is expressed by cancer cells (Pascual et al., 2017) but also by microvascular endothelial cells (Ren et al., 2016), stromal cells (DeFilippis et al., 2012) and immune infiltrating cells (Kitamura et al., 2018). Thus, CD36 is currently studied as an emerging therapeutic target in cancer. Herein we review and discuss recent evidence supporting a key role of CD36 in the progression of different cancer types, the probable tumor progression mechanism, and its implication in the cancer stem cell (CSC) population (Figure 1A).

**TABLE 1:** Reported changes in cancer cellular functions by CD36 activation.

Activating stimuli	Elicited responses	References
CD36 overexpression	Cell proliferation	Drury et al. (2020); Rae et al. (2020); Sakurai et al. (2020); Zhang et al. (2020); Luo et al. (2021)
	Migration, invasion and EMT	Deng et al. (2019); Bort et al. (2020); Drury et al. (2020), Drury et al. (2022); Sakurai et al. (2020); Luo et al. (2021)
	Radioresistance	Park et al. (2019)
	Chemoresistance	Luanpitpong et al. (2019)
	Clonogenicity	Deng et al. (2019); Park et al. (2019); Bort et al. (2020); Rae et al. (2020); Drury et al. (2022)
	CSC-associated immunophenotype	Erhart et al. (2019); Bort et al. (2020)
Fatty Acids (FA)	Cell proliferation	Watt et al. (2019); Zhang et al. (2020); Zhang et al. (2022); Gyamfi et al. (2021)
	Migration, invasion and EMT	Nath et al. (2015); Ladanyi et al. (2018); Deng et al. (2019); Watt et al. (2019); Gyamfi et al. (2021)
	Clonogenicity	Gyamfi et al. (2021)
	CSC-associated immunophenotype	Ghoneum et al. (2020)
	Chemoresistance	Zhou et al. (2009); Woolthuis et al. (2016); Feng et al. (2019); Tse et al. (2020); Zhang et al. (2022)
Oxidized Low Density Lipoproteins (oxLDL)	Cell proliferation	Yang et al. (2021)
	Clonogenicity and CSC-associated immunophenotype	Hale et al. (2014); Yang et al. (2021)

## 2 CD36 in specific cancer types

### 2.1 Breast cancer

Breast cancer has the first place in cancer incidence worldwide and is the fifth place in mortality worldwide by 2020 (Ferlay et al., 2021; Sung et al., 2021). CD36 is overexpressed in breast tumors compared to normal tissue. When analyzed by immunohistochemistry, a minimal or null expression of CD36 was observed in normal tissue samples, whereas 35% of breast tumor samples have high CD36 expression, 46.7% have moderate expression, and only 18.3% low or null expression. The large majority of the high CD36 tumor tissue displays high adipocyte infiltration (Gyamfi et al., 2021), suggesting that CD36 participates in the tumor microenvironment (TME) remodeling.

In HER2^+^ breast cancer patients, high CD36 expression correlates with poor prognosis. In a HER/neu mammary tumor mouse model, CD36 expression is induced by anti-HER2 therapy (lapatinib or trastuzumab) and mammary gland-specific *cd36* knockout suppresses tumor growth and extends survival (Feng et al., 2019). Accordingly, lipid metabolism signaling pathway genes are enriched in HER2^+^ lapatinib-resistant breast cancer cells. In those cells, CD36 is the most highly expressed both at mRNA and protein levels and its knockdown with siRNA induces apoptotic cell death. Furthermore, xenografts from lapatinib-resistant breast cancer cells treated with an anti-CD36 antibody were sensitized to lapatinib (Feng et al., 2020), demonstrating a key role of CD36 in the development and therapy response of HER2^+^ breast tumors.

CD36 expression has been reported in cancer cell lines from different intrinsic subtypes: luminal (Liang et al., 2018; Gyamfi et al., 2021); basal (Castelli et al., 2021; Gyamfi et al., 2021) and HER2-enriched (Feng et al., 2020), but further studies are required to analyze its functional role in those subtypes.

Gyamfi et al. (2021) employed different breast cancer cell lines (BT-483, HCC2218, MCF-7 and MDA-MB-468) co-cultured with adipocytes to analyze the role of CD36 in breast cancer. The interaction between adipocyte and CD36-overexpressing breast cancer cells enhances: 1) lipid droplet (LD) formation and FA accumulation; 2) proliferation; 3) migration and invasion; 4) tumorigenesis in xenograft Balb/c nude mice; 5) FA uptake, which correlates with the activation of an epithelial-mesenchymal transition (EMT) program; and 6) expression of the CSC associated markers CD44, CD133, ALDH, OCT4, and SOX2. The co-culture also promotes the activation of the ERK1/2 and STAT3 signaling pathways with a concomitant increment of the population of cancer cells with a stem cell immunophenotype (CD44^+^/CD36^+^). In agreement, *CD36* knock-down reduced the proliferative, migratory, and invasive characteristics of breast cancer cells and reduced the expression of EMT-regulating transcription factors, as well that of EMT and stemness markers. This evidence demonstrates the role of CD36 in cellular characteristics that are key for breast tumor progression and highlights a special influence of adipocytes in the EMT and stemness induction in CD36^+^ breast cancer cells.

CD36 expression was promoted in tumorspheres from MDA-MB-231 cells exposed to GW6471 (a PPARα selective antagonist). The treatment resulted in: 1) changes in the glucose/lipid metabolism by the decreasing glucose uptake and reducing lactate release; 2) cell cycle arrest with an activation of the intrinsic apoptotic pathway; and 3) reductions in LD with FA and cholesterol accumulation (Castelli et al., 2021). As CD36 has an active role in FA import, it seems to be participating in energy metabolism reprogramming in breast cancer cells.

### 2.2 Hepatocellular carcinoma

Hepatocellular carcinoma (HCC) is the most common primary tumor in the liver, being the sixth most common cancer in incidence and the third cause of cancer death worldwide (Ferlay et al., 2021; Sung et al., 2021). TCGA and Gene Expression Omnibus (GEO) data show that *CD36* mRNA is highly expressed in HCC tissue when compared with normal liver tissues; congruously, HCC cell lines (SMMC-7721, PLC/PRF/5 and HepG2) display high CD36 mRNA and protein levels (Luo et al., 2021). In HCC samples there is a positive correlation between the expression of EMT markers (Vimentin, SNAIL1, ZEB1, ZEB2, TGFB and PORCN) and CD36 expression (Nath et al., 2015). Forced *CD36* overexpression in HCC cell lines (SK-Hep-1 and Huh7) promotes cell proliferation, migration and invasion. The cells show a higher glycolysis and glycolytic capacity with an increased production of lactic acid. *In vivo, CD36* overexpression induces a higher tumor incidence with presence of liver metastasis, which are enriched in CD36^+^ cells. Mechanistically, CD36 activates the Src pathway and its downstream effectors PI3K/AKT in HCC cells; thus, PI3K or AKT inhibitors or the inhibition of the glycolytic pathway reversed the increased cell proliferation and migration (Luo et al., 2021). On the other hand, *CD36* knockdown in HCC cells (SMMC-7721 and HepG2) upregulates FA β-oxidation, activates the lipophagy pathway, reduces hepatic lipid accumulation (Li et al., 2019), impairs cell proliferation, and decreased migration and invasion (Luo et al., 2021). Altogether, these results indicate that CD36-mediated metabolic reprogramming might be vital to HCC development.

CD36 is also involved in the stemness in HCC. Sorafenib-resistant HepG2 and Huh7 cells display increased clonogenicity and tumorigenicity and have a higher expression of efflux transporter ABCB1A and the transcription factors OCT4 and NANOG (Bort et al., 2019). Those cells have increased neutral lipid content accumulation along with a higher expression of enzymes involved in triglyceride and fatty acid synthesis: ATP-citrate lyase, acetyl-CoA carboxylase and fatty acid synthase. Consequently, they have active *de novo* lipogenesis. Increased CD36 expression mediates the higher FA uptake, decreasing β-oxidation by an AMPK mediated mechanism (Bort et al., 2020). As for other cancer types, these findings support the idea that lipid biosynthesis is involved in the stemness maintenance.

### 2.3 Oral squamous cell carcinoma

Oral squamous cell carcinoma (OSCC) arises from the oral cavity and is of the most common type of head and neck cancer (Ali, 2022). CD36 is strongly expressed in invasive OSCC tissues; in contrast, the normal oral epithelium has weak CD36 expression (Sakurai et al., 2020). In a retrospective cohort study with primary diagnosed OSCC, patients with high CD36 expression displayed lower probability of progression-free survival, with only 34% of them alive or recurrence-free at 60 months. The study also demonstrated a 45-fold increased risk of lymph node metastasis in the CD36-high group (Haidari et al., 2021).

In multiple OSCC cell lines (HSC-1, HSC-3, HSC-4, and Ca9-22), CD36 expression correlates with Ki-67, PDGFRβ, and with a low E-cadherin expression (Sakurai et al., 2020), suggesting that CD36 participate in cell proliferation and migration. Accordingly, CD36^+^ cells have a higher migratory activity than CD36^−^ (Sakurai et al., 2020) and forced *CD36* overexpression in OSCC cell lines (SCC-25, VDH-00 and JHU-029) or patient derived cells upregulates metastatic associated genes and increases their potential to metastasize to the lymph nodes (Pascual et al., 2017).

The FA β-oxidation is crucial for the establishment of metastasis by CD36^+^ cells. The subpopulation of OSCC cells with the CD36^+^/CD44^bright^ immunophenotype expresses higher levels of enzymes involved in FA β-oxidation. High-fat feeding of NGS mice or *ex vivo* exposure of cancer cells to palmitic acid (PA) increases the size and frequency of lymph node metastases in OSCC xenotransplants (Pascual et al., 2017). On the opposite, FACS-sorted CD36^−^ do not generate lymph node metastasis and *CD36* expression knock-down by short-harpin RNA (shRNA) reduces the metastatic burden. Although cells with a mutant CD36 (CD36-K164A) can generate some lymph node metastasis, the lesions have cells with increased content of LD (Pascual et al., 2017), indicating that CD36 is essential for the metabolic reprogramming coupled to the adapting to the metastatic niche. Furthermore, CD36^+^/CD44^bright^ cells display a tenfold increase in metastatic potential compared with CD44^bright^ population and generate metastatic lesions that recapitulate molecular and cellular heterogeneity found tumors from the parental cells (Pascual et al., 2017), suggesting that CD36 may be a marker for the population of metastasis-initiating cells.

### 2.4 Leukemias and lymphomas

Leukemia is characterized by the abnormal proliferation of blood cells in the bone marrow and blood forming organs (Roy et al., 2014). In acute myeloid leukemia (AML) patients, CD36 was highly expressed in tumors with an advanced stage. CD36 expression correlates with unfavorable cytogenetics, shorter overall survival, and a shorter leukemia free survival (Abd El-Aziz et al., 2013).

In AML, CD36 cooperates with soluble signals from the TME to promote cancer progression and resistance to chemotherapy. AML cells pretreated with IL-6 show increased lipid accumulation, enhanced uptake of FA, and enhanced CD36 expression at mRNA and protein level (Zhang et al., 2022). Accordingly, AML patients with high IL-6R expression show chemoresistance and altered lipid metabolism (Zhou et al., 2009; Tse et al., 2020). The signaling elicited by IL-6 triggers STAT3 activation (Mauer et al., 2015), enhancing tumorigenesis and chemoresistance (Zhou et al., 2009). Reduction of FA uptake by CD36 inhibition represses the IL-6-induced resistance to cytotoxic drugs. Similarly, CD36 knockdown decreases FA uptake and increases chemotherapeutic-induced apoptosis, even in the presence of IL-6, demonstrating that CD36 is involved in chemoresistance induction in AML cells. *STAT3* knockdown decreases CD36 expression and FA uptake but increases apoptosis, showing that IL-6 induced chemoresistance is dependent on STAT3, which in turn, promotes CD36 expression by regulating STAT3 activation (Zhang et al., 2022).

Along with CD36, apoliprotein C2 (APOC2) is highly expressed in AML patient samples. Using HEK293T cells, it has been shown that APOC2 coimmunoprecipitate with CD36 (Zhang et al., 2020). The simultaneous overexpression of APOC2 and CD36 in THP-1 or MOLM-13 AML cell lines enhances cell proliferation, triggered by ERK and LYN phosphorylation (a downstream CD36 target), in comparison with APOC2 or CD36 overexpression alone. *CD36* knockdown by shRNAs causes a decrease in p-ERK protein levels even in the APOC2 overexpressing cells, suggesting a crucial role of CD36 in ERK activation. *In vivo APOC2* and *CD36* knockdown reduces leukemia burden and engraftment to bone marrow, spleen, and peripheral blood (Zhang et al., 2020); therefore APOC2-CD36 signaling may be an attractive therapeutic target.

Mantle cell lymphoma (MCL) is an aggressive non-Hodgkin lymphoma subtype with the worst prognosis, poor response to chemotherapy, and high rate of drug resistance development (Zaja et al., 2014). MCL patients with the highest CD36 expression have shorter overall survival (Luanpitpong et al., 2019). In MLC, Bortezomib (BTZ) is combined with cytotoxic chemotherapy to get a higher response rate (Yazbeck et al., 2018). BTZ-resistant MLC cells have increased cellular lipids content and upregulation of CD36 protein on cell surface and intracellular compartment. The overexpression of CD36 results in accumulation of LD and decreased apoptosis. In agreement, the inhibition of CD36 in the same cells sensitizes them to BTZ induced apoptosis (Luanpitpong et al., 2019). These results advises the use of CD36 inhibition in combination therapy to treat drug resistant MCL and suggest that CD36 expression could be considered predictive of therapeutic response.

CD36 seems to also play an important role in the biology of B-lymphoblastic leukemia (B-LL) and chronic lymphocytic leukemia (CLL), although little evidence exists. In pediatric B-LL samples from Children’s Health of Atlanta, the expression of CD36 correlates with poor outcome. The samples from patients with CD36^+^ B-LL cells have a reduced event-free survival and reduced overall survival compared with CD36^−^ patients (Newton et al., 2017). In CLL, CD36 expression is higher in tumor samples than in healthy individuals’ samples. CCL cells store lipids in vacuoles and have active the metabolic pathways by which adipocytes and myocytes produce energy, a process that may be dependent on CD36. As in AML cells, downregulation of STAT-3 in CLL cells by shRNA reduces CD36 expression (Rozovski et al., 2018).

Even though tyrosine kinase inhibitors are highly effective treatments for leukemias, the disease is not completely cured because the leukemia stem cells (LSC) subpopulation is partially resistant to these drugs (Jiang et al., 2007). LSC can co-opt their TME, like adipose tissue, to support their unique metabolic needs and promote their survival and growth (Woolthuis et al., 2016). Gonadal adipose tissue (GAT) serves as a reservoir for LSC and helps to turn on the FA metabolism in a subpopulation of LSC that express CD36 and have high fatty acid oxidation (FAO). Although the CD36^+^ cells have metabolic characteristics that resemble those of quiescent hematopoietic stem cells, CD36 expression was undetected in normal stem/progenitor populations. CD36^+^ LSC subpopulation is less chemo-sensitive than their counterpart CD36^−^ LSC when exposed *ex vivo* to different chemotherapeutic drugs*.* Those findings were corroborated *in vivo* in a murine model of AML where the CD36^+^ LSC in the bone marrow and in GAT were enriched after chemotherapy. *CD36* knockout reduced FA uptake and FAO, decreased leukemic burden in GAT, and increased the response to chemotherapeutic drugs in leukemic mice, indicating that CD36 enhances leukemic colonization of GAT and contributes to chemo-resistance of LSC. Similar results were found at least in four of eight primary human specimens of chronic myeloid leukemia (CML) or AML specimens (Woolthuis et al., 2016). This study: 1) proposes the existence of metabolic heterogeneity in LSC; 2) finds a role of CD36^+^ LSC in tumor progression; and 3) suggests that FA metabolism modulation may contribute to eradicating LCS.

### 2.5 Bladder cancer

Bladder cancer is the most common neoplasm of the urinary tract, and there are two types with different characteristics at the molecular level: in 75% of cases, it is confined to the mucosa and is called non-muscle-invasive bladder cancer (NMIBC), whereas the other cases correspond to muscle-invasive bladder cancer (MIBC) (Dobruch and Oszczudłowski, 2021; Jeong et al., 2021). NMIBC tumor samples show increased expression of the FA transporters FATP4, CD36 and ACSL1 (Jeong et al., 2021). In MIBC-type bladder cancer, CD36 expression correlates to greater depth of tumor invasion (pT stage) and advanced stages of the disease (pT3b-T4) (Pardo et al., 2022).

CD36 expression is important in the biology of the CSC population from bladder cancer tumors. Silencing of *CD36* in human bladder cancer cells reduces the clonogenicity and the expression of stemness markers (ALDHA1, CD44, KLF4 and Nanog) induced by oxLDL exposure. In agreement, *CD36* knockdown reduces tumor growth in xenotransplants, the pro-tumorigenic effect of the high in fat and cholesterol diet, and the tumor expression of stemness markers (Yang et al., 2021). The evidence demonstrates that CD36 participates in maintaining stemness and tumor growth progression and that oxLDL, not only FA as reported for other cancer cell types, might trigger relevant CD36 mediated signals.

### 2.6 Glioblastoma

Glioblastoma (GBM) is the most aggressive and the most frequent malignant brain tumor in adults (DeAngelis, 2001). Data collected by Liang et al. and analyzed by Hale et al., shows that CD36 expression at mRNA and protein levels correlates with poor prognosis of GBM patients, pointing CD36 as a prognosis biomarker for patient survival (Liang et al., 2005; Hale et al., 2014).

Human GBM are highly vascularized tumors that rely on new blood vessels formation for growth (Jain et al., 2007). Thus, antiangiogenic therapies have been employed to target GBMs, but they resulted in the promotion of tumor invasion and recurrence (Pàez-Ribes et al., 2009; Keunen et al., 2011). TSP-1 is an anti-angiogenic protein that elicits its effects through its three type-1 repeats (3TSR). 3TSR induce apoptosis in endothelial cells through the interaction with CD36, which activates p59fyn/p39/caspase-3 mediated apoptosis or upregulates TRAIL death receptors DR4 and DR5 (Jiménez et al., 2000; Ren et al., 2009). Since CD36 is also expressed in a subset of GBM cells, engineered mesenchymal stem cells expressing TSR3 have been employed to sensitize GBM cells to TRAIL-mediated apoptosis through activation of CD36 signaling and upregulation of DR4/DR5 TRAIL receptors (Choi et al., 2015).

CD36 is expressed in GBM CSC, where it helps in stemness maintenance. CD36^high^ cells had elevated capacity to form tumorspheres in culture along GBM specimens and CD36 is expressed in subpopulations positive for other CSC markers like integrin α6, CD133 and SOX2 (Hale et al., 2014). Multidimensional stemness marker analysis identified that the CD44^+^/CD133^+^/ITGA6^+^/CD36^+^ immunophenotype is consistently useful to enrich in cells with stem cell characteristics from diverse sources, and the combination of CD44, CD133, ITGA6 and CD36 expression identifies tumors of patients with significantly shorter survival (Erhart et al., 2019). *CD36* knockdown by siRNA across multiple GBM xenograft specimens impacts on the CSC status by 1) reducing integrin α6 expression; 2) attenuating tumorspheres formation; 3) decreasing stem cell frequency; 4) abrogating tumor initiation capacity; and 5) decreasing key CSC maintenance signaling pathways like SOX2, phospho-AKT and phospho-STAT3 (Hale et al., 2014). Furthermore, activation of CD36 signaling by oxLDL increases proliferation of the CSC population (Hale et al., 2014), demonstrating that the CSC pool expansion associated with poor prognosis is partially mediated by CD36 recognition of oxLDL.

### 2.7 Prostate cancer

Prostate cancer (PC) is the most diagnosed and the second leading cause of death among men (Siegel et al., 2022). PC patients with high saturated fat levels of dietary intake increase their risk of mortality (Epstein et al., 2012; Richman et al., 2013) and glucose uptake, *de novo* lipogenesis, FA uptake and FA storage are increased in malignant prostate tissue (Watt et al., 2019), indicating that lipid metabolism promotes PC progression. Accordingly, high *CD36* gene expression is associated with reduced relapse-free survival and increased incidence in metastases in the PC TCGA cohort (Watt et al., 2019).

Exposure of PC human cell lines (PC3 and LNCaP) to FA promotes FA uptake and enhances cell proliferation. Conversely, *CD36* knockdown by shRNA reduces: 1) free-FA uptake and oxidation; 2) FA incorporation into complex lipids; 3) cell proliferation; and 4) migration in response to free-FA. The re-expression of CD36 into knocked down cells restores FA uptake and proliferation rates into basal levels compared to parental PC cell lines (Watt et al., 2019). This evidence is in agreement with the fact that periprostatic adipose tissue can supply FA for supporting PC progression (Taylor et al., 2015; Lo et al., 2016). In xenotransplants formed by PC cells with *CD36* knockdown, FA uptake from TME is impaired, reducing lipid biosynthesis, activation of oncogenic lipid signaling pathway, and attenuating tumor growth (Watt et al., 2019). Thus, the CD36-mediated lipid metabolism promotes PC progression.

### 2.8 Gynecological malignancies

Ovarian cancer (OvCa) is the fifth most common cancer and one of the most lethal cancers in females (Siegel et al., 2022). CD36 expression is upregulated in primary human OvCa and in human OvCa visceral metastases (Wang et al., 2016). CD36 might be mediating the paracrine tumor-growth stimulation by adipocytes, since adipocytes promote OvCa growth and metastasis through the provision of FA (Nieman et al., 2011; Nieman et al., 2013). The co-culture of human primary adipocytes (HPAs) with OvCa cell lines (SKOV3ip1, HeyA8 or OVCAR-5) induces in cancer cells: 1) CD36 expression at mRNA and protein levels; 2) FA uptake and increased intracellular cholesterol content; 3) LD accumulation; and 4) changes in OvCa transcriptome characterized by the induction of a pro-inflammatory cytokine profile, oxidative stress, and the activation of pathways that regulate lipid and cholesterol synthesis (Ladanyi et al., 2018). *CD36* silencing by shRNA in SKOV3ip1 cells reduced: 1) CD36 constitutive and adipocyte-induced expression; 2) the FA uptake and intracellular cholesterol content even at HPA stimuli; 3) LD accumulation; 4) baseline and adipocyte-stimulated invasion and migration; 5) anaerobic glucose metabolism while enhancing glucose oxidation; and 6) *in vivo* tumorigenesis in nude mice as well as the number of metastatic nodules (Ladanyi et al., 2018).

The CD36 role in the ovarian CSC population has recently started to be described. Analysis of the transcriptome of CSCs from the OVCAR3 cell line shows that the *CD36* mRNA is significantly increased along with carboxylases that are involved in the FA biosynthesis. Ovarian CSCs display a positive correlation between CD36 expression and that of lipid metabolic enzymes and stemness marker OCT4 (Ghoneum et al., 2020). However, further studies are required to describe the functional implications of CD36 in CSCs from ovarian tumors.

In advanced cervical cancer, CD36 expression is correlated with poor tumor differentiation, EMT markers expression, and positive lymph node metastasis. Thus, high CD36 expression has been proposed as a biomarker for unfavorable prognosis. CD36 is expressed in human cervical cancer cell lines (C33a, Hce1, HeLa and SiHa cell lines). Treatment of cervical cancer cells with TGF-β, a classic EMT inducer, promotes CD36 expression, indicating a link between EMT activation and CD36 expression. The knockdown of CD36 by siRNA in cervical cell lines: 1) inhibits cell migration; 2) diminishes invasion ability; 3) attenuates colony formation; 4) increases the apoptosis rate; and 5) changes EMT-associated morphology and protein expression (Deng et al., 2019). In agreement, forced CD36 overexpression in cervical cancer cells promotes proliferation and enhances cell migration and invasion (Yang et al., 2018; Deng et al., 2019). Furthermore, *CD36* overexpression favors *in vitro* colony formation, promotes EMT (Deng et al., 2019) and activates the Src/ERK1/2 signaling pathway (Yang et al., 2018). *In vivo,* CD36 overexpressing cells display increased tumor growth kinetics and metastatic ability (Yang et al., 2018; Deng et al., 2019).

### 2.9 Colorectal cancer

Colorectal cancer (CRC) is the third cause of incidence and the second leading cause of cancer-related death worldwide by 2020 (Ferlay et al., 2021; Sung et al., 2021). CRC tissue microarray analysis showed CD36 levels were significantly higher than normal colon mucosa and CD36 expression is increased in CRC liver and lung metastases (Drury et al., 2022). Data from The Human Protein Atlas show that *CD36* high mRNA level is associated with reduced 5-year survival in CRC patients. However, *CD36* mRNA is significantly lower in CRC cancer tissues in the TCGA database (Drury et al., 2020) or in studies available in GEO (Zhang X. et al., 2019). To address the discrepancy, Drury et al. (2020) studied CD36 protein expression in CRC primary tumors, finding that it can be similar or higher to the level in normal colon mucosa, whereas the glycosylated form of CD36 is higher in CRC liver metastasis compared to normal liver or colon mucosa. Analysis of CRC patient-derived xenograft tumors from primary and metastatic CRC tumors demonstrated that the expression of glycosylated CD36 is associated with metastatic tumors.

Mechanistic studies of the role of CD36 have been performed in CRC models. *CD36* overexpression in CRC cell lines (HCT116, HT29, HT29LuM3) resulted in: 1) upregulation of cell proliferation by high survivin expression and a decreased caspase-3 and PARP cleavage; 2) enhanced invasion ability; and 3) increased colony formation and diameter (Drury et al., 2020). Opposite effects are generated by forced reduction of *CD36* expression by shRNA. The *CD36* overexpressing cells displayed higher frequency of orthotopic tumors and formed more metastatic nodules than control cells (Drury et al., 2022), whereas CD36 knocked-down cells formed significant lower lung metastasis after tail vein injection, suggesting that CD36 promotes the expansion of tumor- and/or metastasis-initiating cells. Moreover, CD36^high^ cells isolated from CRC patient-derived xenografts have high survivin expression and formed larger tumors than CD36^low^ cells derived from the same tumor (Drury et al., 2022), indicating that CD36 also plays a role in CRC cell survival and/or proliferation. On the other hand, Fang et al. found that CD36 protein expression is absent or weak in human CRC samples and associated negative CD36 signal to poorer overall survival (Fang et al., 2019). Thus, further studies using standardized methods and reagents are required to clarify whether CD36 protein expression can be prognostic in CRC.

CD36 may also be participating in radiotherapy (RT) resistance of CRC cells. In HCT116 cells, JAK2 expression was elevated after RT, leading to the activation of STAT3 which causes limited RT-induced apoptosis and enhanced clonogenic potential (Park et al., 2019). *CD36* was upregulated -among other genes- by RT and JAK2 silencing abolished such effect (Park et al., 2019), demonstrating that *CD36* is a target gene of the JAK2/STAT3 pathway in CRC cells. As CD36 plays a role in stemness (see above), this receptor may participate in the survival of the CSC population to RT but more studies are needed to establish a precise relation.

In contrast, it has been reported that CD36 plays an opposite role in CRC progression. CD36 overexpression in SW480 or LoVo cells significantly: 1) inhibited cell proliferation; 2) inhibited colony formation; 3) resulted in cell cycle arrest and increased apoptosis; 4) decrease in the migratory and invasive ability; and 5) induced a metabolic shift from aerobic glycolysis to oxidative phosphorylation. *CD36* knockdown by shRNA in RKO and Caco2 cells promoted: 1) proliferation; 2) colony formation; 3) cell cycle progression; 4) migration and invasion and 5) glycolytic repression (Fang et al., 2019). These results indicate that CD36 has a tumor-suppressive role in CRC cells, differing from those from Park et al. Thus, more research is needed to elucidate the CD36 role in CRC development and progression.

## 3 CD36 in tumor stroma cells

Endogenous normal cells are frequently recruited into the tumor (Kidd et al., 2012). Stroma cells like fibroblast, adipocyte, endothelial, perivascular, and immune cells influence tumor progression and therapy response (Ricci-Vitiani et al., 2010; Cheng et al., 2013). In *Cd36* ^−/−^ mice receiving different cancer cell implants (murine Lewis lung carcinoma LCC cells, murine B16F10 melanoma cells, Hepa1-6 hepatoma cells, CT26 or MCA-38 colon cancer cells) tumor growth is reduced, and the number of metastases is decreased compared to WT mice (Al-Khami et al., 2017; Yang et al., 2022). Those effect are associated with impaired cancer cell proliferation and angiogenesis (Al-Khami et al., 2017; Yang et al., 2022), demonstrating the importance of CD36 expression in the host for tumor progression.

In agreement, intratumoral Treg cells upregulate CD36 expression and activate the PPAR-β pathway and lactate metabolism to survive in the tumor microenvironment. Treg cells from non-small-cell lung carcinoma, melanoma and CRC display increased FA uptake, high lipid content and CD36 upregulation compared with Tregs from other tissues (Wang et al., 2020). The number of intratumoral Treg cells is reduced in Treg-specific *Cd36*-deficient mice with tumor grafts, which is associated with an increased amounts of CD8^+^ and CD4^+^FoxP3^-^ tumor-infiltrating lymphocytes (TILs) and reduced tumor growth (Wang et al., 2020). Frank and collaborators reported that apoptotic breast cancer cells release miR-375, which binds to low-density lipoproteins and can be uptake by macrophages *via* CD36 receptor (Frank et al., 2019). The uptake by macrophages increases their migration, promotes tumor infiltration (Frank et al., 2019) and might direct them into a pro-tumoral phenotype (Huang et al., 2014; Frank et al., 2019).

On the contrary, CD36^+^ fibroblast cause inhibition of breast cancer cell proliferation by secreting proteins like SLIT3, PENK and FBLN1 (Jabbari et al., 2021; Jabbari et al., 2022), indicating that CD36^+^ fibroblasts may play an anti-tumoral role. In a breast cancer murine model, diet-induced obesity activates the Lysophosphatidic acid/protein kinase D (PKD-1) signaling pathway, downregulating *CD36* expression in the tumor endothelium, which could be a key step in initiating the microvascular remodeling (Dong et al., 2017).

The above evidence indicates that: 1) CD36 has an important role in the bidirectional communication between cancer and stroma cells; 2) CD36 could be playing pro- or anti-tumoral roles in different cellular contexts; and 3) the effects on stroma cells should be considered in CD36 targeting. We anticipate that the characterization of the precise effects of CD36^+^ stroma cells in tumor biology will be a matter of intense study in the following years.

## 4 Strategies to inhibit CD36 function

Given that loss-of-function studies described above demonstrated that CD36 is a valid target for altering cancer progression, multiple groups have proposed its pharmacological inhibition. In general, three strategies have been reported in the literature for inhibiting the function of CD36 as a lipid transporter: 1) the use of monoclonal antibodies directed to the ligand-binding sites; 2) the use CD36-binding small molecules; and 3) the inhibition of protein expression (Table 2).

**TABLE 2:** Strategies for blocking CD36 and their reported effects.

Type of inhibitior	Reported effects	References
Blocking antibodies	Reduction in the FA and oxLDL uptake	Pascual et al. (2017); Jiang et al. (2019); Watt et al. (2019); Wang et al. (2020); Aznar et al. (2021); Xu et al. (2021); Yang et al. (2021)
	Inhibition of the TSP-1 interaction	
	Tumor growth inhibition	
	Decrease in size or remission of metastases; decrease of stemness markers and in the population percentage of ALDH1A1, CD44, KLF4 and NANOG	
	Decreased clonogenicity and cell proliferation	
	Promoted apoptosis in intratumoral Treg cells	
	Increased tumor infiltration of CD8^+^ T cells	
	Reduced the frequency of intratumoral Treg cells	
Small-molecule antagonists	Reduction in the tumorspheres size	Bao et al. (2012); Sp et al. (2018); Frégeau et al. (2020); Vazquez et al. (2020)
	Decreased cholesterol and cholesteryl-esters accumulation	
	Decreased uptake of modified LDL (Dil-acLDL)	
	Decrease in atherosclerotic lesions *in vivo*	
CD36 expression inhibitors	Diminished oxLDL uptake and reduction of the transformation of macrophages into foam cells	Lian et al. (2008); Dai et al. (2014); Li et al. (2015); Yang et al. (2016)

### 4.1 The CD36-targeting antibodies

There are currently several commercially available anti-CD36 antibodies. Those antibodies have been used as a proof-of-concept that CD36 blockage can be efficiently achieved to alter tumor progression. In a seminal work, Pascual et al. evaluated the effect of two CD36 inhibitory antibodies in an *in vivo* model of OSCC: clone JC63.1 -a murine IgA reported as a blocker of oxLDL transport- and clone FA6-152 -a murine IgG_1_ that blocks fatty acid transport and TSP-interaction-(Mwaikambo et al., 2006). Antibody-treatment of NGS mice orthotopically inoculated with OSCC inhibits the formation of lymph node metastasis without affecting the primary tumor onset and reduces the size of established metastases, generating a complete metastatic remission in 15% of the mice. To analyze the toxicity of the treatments, authors analyzed weight of the individuals, liver and kidney histology, hepatic transaminases, hemoglobin, and blood cell count, without finding significant changes (Pascual et al., 2017), suggesting that, despite its ubiquitous expression, CD36 blockage does not alter body homeostasis.

CD36-targeting antibodies are also effective in other models of cancer. For example, JC63.1 decreases bladder cancer cells stemness and malignancy induced *in vitro* by oxLDL, as well as *in vivo* tumor growth promoted by high-fat high-cholesterol diet (Yang et al., 2021). JC63.1 inhibits the onset of metastasis in nude mice xenotransplanted with gastric cancer cells (Jiang et al., 2019). Antibody-mediated CD36 blockage reduces viability of PC cells and clonogenic survival in PC-derived organoids (Rae et al., 2020) and inhibits *de novo* lipogenesis in human PC-derived organoids (Watt et al., 2019). In NSG mice harboring PC patient-derived xenografts with high uptake of fatty acids and CD36 expression, JC63.1 reduced tumor volume but had no significant effect on the weight of individuals or the concentration of blood lipids (Watt et al., 2019). CD36 targeting with polyclonal antibodies induce selective killing of primitive CD34^+^/CD38^low^ CML cells (Landberg et al., 2018).

Furthermore, CD36 targeting may be affecting not only CD36^+^ cancer cells but also stromal or infiltrating tumor cells. In a murine model of melanoma, JC63.1inhibits tumor growth by promoting TNF expression in CD8^+^ TILs and reducing the frequency of intratumoral Treg cells (Xu et al., 2021). Treg cells located in melanoma tumors have upregulated *CD36* expression and enhanced FA uptake compared with Treg cells from other tissues. In mice treated with JC63.1, tumors show delayed growth kinetics and reduced lipid uptake by intratumoral Treg cells, a phenotype that mimics lineage-specific genetic ablation of CD36 in Tregs (Wang et al., 2020). The antibody promotes apoptosis in intratumoral Treg cells, increases tumor infiltration of CD8^+^ T cells, and favors the production of antitumor cytokines in CD8^+^ and CD4^+^ TILs.

Because of the above evidence, the interest in developing human or humanized anti-CD36 antibodies with translational potential has grown in recent years. For example, the company Ona therapeutics has developed several anti-CD36 antibodies as anti-tumor candidates (Aznar et al., 2021). Clone 1G04 is an IgG_1_ that partially blocks FA uptake in cancer cells and reduces tumor growth in breast and colon adenocarcinoma isotransplants, as well as in xenotransplants of lung cancer cells. As expected from the pro-metastatic role of CD36, the antibody also decreased the number of metastases in the liver and lung (Aznar et al., 2021). The clone Ona-0-v1 is another anti-CD36 with *in vivo* activity in mouse models of ovarian, CRC, and oral cancer. For example, in xenotransplants of CRC cells, Ona-0-v1 significantly decreases tumor size and reduces 60–80% liver and lung metastases (Aznar et al., 2021). In summary, anti-CD36 antibodies blocking the FA and the oxLDL transport *in vivo* models in different types of cancers have an antitumor, antimetastatic effect and decreased the CSC (Figure 1B).

### 4.2 Small molecules as CD36 antagonists

Another strategy to modulate CD36 function is the use of small molecules that inhibit receptor activity. Several antagonists have been identified for such a purpose, mainly in efforts to discover effective treatments for atherosclerosis, where CD36 is crucial for pathogenesis. The activity of some of those inhibitors has been studied in cancer models, providing further support to the idea that targeting CD36 as a valid strategy for cancer treatment.

Nobiletin is a flavonoid isolated from citrus peel. Through molecular docking, it was shown that Nobiletin´s atoms 11–20 bind to the extracellular domain of CD36 and inhibits CD36 binding to TSP-1 *in vitro* (Sp et al., 2018). In MCF-7 breast cancer cells stimulated with PA, Nobiletin mimics the effects of *CD36* knock-down, decreasing tumorsphere formation, the expression of stemness markers (SOX2, OCT4 and NANOG), STAT3 phosphorylation, and NF-kB activation. Inactivation of the CD36/STAT3/NF-κB signaling axis by Nobiletin reduces the expression of CD36 by a feedback loop since *CD36* is a target gene of both STAT and NF-κB (Sp et al., 2018).

Nitro-oleic acid (NO2-OA) is a partial PPARγ agonist that increases CD36 expression in a dose-dependent manner. However, in RAW264.7 cells, a 15 min pre-incubation with NO2-OA reduces the accumulation of cholesterol and cholesteryl-esters and inhibits modified low-density lipoprotein (mLDL) binding and uptake. NO2-OA interacts with CD36 in immunoprecipitation assays and the binding mode has been predicted by molecular docking where NO2-OA binds to the Lys164 to CD36 region, a key residue in lipid transport (Vazquez et al., 2020).

Sulfo-N-succinimidyl oleate (SSO) has been also reported as CD36 antagonist (Urso and Zhou, 2021). CD36 inhibition by SSO in HepG2 and Hep3B diminish the EMT phenotype, upregulates E-cadherin expression, and reduces the migration rate of cells treated with PA (Nath et al., 2015). SSO also decreases cellular proliferation and increases cleaved caspase-3 levels in primary CRC cells and reduces tumor growth in xenografts (Drury et al., 2020). Vázquez et al. suggested that SSO and Nolbiotin share the same binding site (Vazquez et al., 2020).

Azapeptides are synthetic molecules analogous to growth hormone-releasing peptide-6 (GHRP-6) that bind to the oxLDL binding site on CD36. MPE-001 and MPE-003 azapeptides are effective in reducing sinus and aortic arch lesions in a mouse model of atherosclerosis (high-fat high cholesterol fed ApoE^−/−^ mice) (Frégeau et al., 2020). However, their effects in cancer cells have not been studied.

Salvianolic acid B (SAB), rosmarinic acid, and sodium danshensu, were identified as CD36 inhibitors using an ELISA–like high-throughput screening (Wang et al., 2010). Interaction between SAB and recombinant CD36 was demonstrated by Surface Plasmon Resonance analysis. *In vitro*, SAB blocked the binding and absorption of mLDL in RAW 264.7 cells (Wang et al., 2010) and the uptake of Dil-acLDL in phorbol-12-myristate-13 acetate (PMA)-stimulated THP-1 and RAW 264.7 cells (Bao et al., 2012). Furthermore, SAB decreases the levels of *CD36* mRNA in THP-1 cells and RAW 264.7 stimulated with oxLDL (Bao et al., 2012), which can be partially explained by the previously reported (Sp et al., 2018) feedback loop activated by CD36-ligand binding.

Finally, 2-methylthio-1,4-naphthoquinone (MTN) is a specific CD36 inhibitor (Müller et al., 2004) with anti-cancer activity in GBM. MTN reduces: 1) tumorsphere formation capacity; 2) the CSC frequency; 3) the activation of CSC signaling pathways; and 4) survival of GBM CSC populations (Hale et al., 2014).

Despite the promising results of the molecules described above, further studies are required to clarify: 1) their binding mode to CD36 and the structural mechanisms involved in the inhibition; 2) their off-target effects; and 3) their toxicological profiles. This information may lead to the development of inhibitors with increased affinity and specificity.

### 4.3 Modulation of CD36 expression

Pharmacological silencing of CD36 has also shown positive results in cancer models. Myricetin is a flavonoid that interferes with the PPARγ pathway and therefore negatively regulates the expression of CD36. Myricetin induces a 5-fold decrease in *CD36* mRNA expression in PMA-differentiated U937 cells; consequently, the cells have a reduced capability to uptake of oxLDL (Lian et al., 2008).

Intermedin (IMD) reduces the *in vitro* transformation of mouse peritoneal macrophages into foam cells by reducing oxLDL uptake (Dai et al., 2014). Using macrophages isolated from atherosclerotic or peritoneal lesions of apoE^−/−^ mice treated with IMD for 6 weeks, it was shown that the decrease in oxLDL uptake is caused by the reduction in *CD36* mRNA expression (Dai et al., 2014). Similar effects have been observed with Paeonol, an antioxidant isolated from the root bark of *Paeonia suffruticosa* that promotes apoptosis in cancer cells (Zhang L. et al., 2019) and Amentoflavone, a biflavonoid isolated from *Selaginella tamariscina* that reduces proliferation, invasion, and drug resistance in different types of cancer (Xiong et al., 2021). In macrophage-like cells stimulated with oxLDL and treated with Paneolol or Amentoflavone, lipid accumulation is reduced (Li et al., 2015; Zhuang et al., 2021). Paneolol inhibits CD36 expression through modulation of the c-Jun-AP-1 pathway (Li et al., 2015), but Amentoflavone suppresses the PPARγ/CD36 signaling pathway (Zhuang et al., 2021). Simvastatin, an FDA-approved HMG-CoA reductase inhibitor, also decreases oxLDL uptake in mouse peritoneal macrophages by down-regulating the expression of CD36 (Yang et al., 2016).

As mentioned for small-molecules antagonists, the inhibitors of CD36 expression require further studies to validate their specificity and characterize their adverse effects in order to have translational potential. In the meantime, we consider that this subtype of drugs should be used cautiously as experimental tools for studying CD36 biology.

## 5 Conclusion

In several types of cancer, there is a positive correlation between tumor CD36 expression and poor clinical outcome (Abd El-Aziz et al., 2013; Hale et al., 2014; Deng et al., 2019; Luanpitpong et al., 2019; Haidari et al., 2021; Jeong et al., 2021; Luo et al., 2021; Yang et al., 2021; Pardo et al., 2022). Mechanistically, CD36 participates in tumor development by favoring the lipid intake in cancer cells and promoting a switch in lipid metabolism to settle the increasing energetic demand of the tumor.

CD36 has been related to cell proliferation and tumor growth kinetics in several types of cancers (Deng et al., 2019; Watt et al., 2019; Drury et al., 2020; Ghoneum et al., 2020; Sakurai et al., 2020; Gyamfi et al., 2021; Haidari et al., 2021; Luo et al., 2021; Drury et al., 2022). There is also evidence of a positive correlation between CD36 expression/activation and EMT induction in cancer cells, where CD36-mediated FA uptake/accumulation and the consequent energetic reprogramming are key for the acquisition of a more aggressive phenotype. However, the CD36 downstream signaling pathways activated in each tumor type remain to be described. The exception is the AKT/ERK1/2/STAT3 pathway, which is affected by CD36 silencing or blocking in breast cancer (Sp et al., 2018; Gyamfi et al., 2021), HCC (Luo et al., 2021), leukemia (Zhang et al., 2020), GBM (Hale et al., 2014), cervical cancer (Yang et al., 2018), and CRC (Park et al., 2019). Identification of the signal transduction pathways activated by CD36 will allow the rational design of combination therapies. For example, combination of CD36 and AKT inhibitors reduce cell proliferation in HCC (Luo et al., 2021).

CD36 is expressed and has a functional role in the CSC-pool. For example, CD36 is coexpressed with the stemness markers CD44^+^/CD133^+^/ALDH^+^ in breast cancer (Gyamfi et al., 2021), with CD44^bright^ in OSCC (Pascual et al., 2017), and with CD44^+^/CD133^+^/ITGA6^+^ in GBM (Hale et al., 2014; Erhart et al., 2019). Furthermore, CD36 expression promotes radio and chemoresistance in breast cancer (Feng et al., 2020), AML (Woolthuis et al., 2016; Zhang et al., 2020), MCL (Zaja et al., 2014), and CRC (Park et al., 2019), which are known to be (at least partially) mediated by CSCs. In agreement, CD36 blockage reduces the fraction of CSCs (Hale et al., 2014; Pascual et al., 2017; Bort et al., 2019; Erhart et al., 2019; Bort et al., 2020; Ghoneum et al., 2020; Gyamfi et al., 2021; Luo et al., 2021; Yang et al., 2021; Drury et al., 2022).

Thus, CD36 can become an additional biomarker to distinguish the CSC population or to identify subpopulations of CSC with specific functions. For example, the facts that CD36 inhibition reduces EMT in breast cancer (Gyamfi et al., 2021), HCC (Nath et al., 2015), and cervical cancer (Deng et al., 2019), and drastically affects metastasis with a modest/null effect in primary xenotransplants (Pascual et al., 2017) suggest that either the CD36-mediated metabolic switch is particularly important in the metastatic niche and/or CD36 plays a differential role in metastasis-initiating and tumor-initiating cells. In any case, the molecular mechanisms by which CD36 promotes stemness in the primary and metastatic sites constitute opportunities for future research.

Furthermore, CD36 is relevant in the communication between cancer and non-cancerous cells within tumors. For example, normal cells recruited into the TME (adipocytes, cancer associated fibroblast, macrophages) can be the source of FA that activate CD36 in cancer cells (Pascual et al., 2017; Ladanyi et al., 2018; Watt et al., 2019; Gyamfi et al., 2021; Luo et al., 2021).

Recently, Yang and collaborators described the metabolic crosstalk between tumor cells and macrophages in liver metastases, in which CD36 is upregulated in the metastasis-associated macrophages that participate in creating an immunosuppressive TME (Yang et al., 2022). Another example is the overexpression of CD36 in Treg cells in melanoma, which favors the dysfunction of TIL (Wang et al., 2020; Xu et al., 2021). This evidence calls for the study of the role of CD36 in other normal cells located in the TME and suggests that CD36 targeting may elicit anti-tumor responses by multiple mechanisms.

The role of CD36 in tumor progression supports its importance as a therapeutic target. It has been demonstrated that CD36 inhibition phenocopies CD36 knockdown, decreasing tumor growth, metastatic dissemination and stemness. Thus, CD36 inhibitors could become anti-cancer therapies. However, there are still many open questions regarding such inhibitors. For example: what are the epitopes recognized by anti-CD36 antibodies? What are the binding sites and binding modes of small-molecule inhibitors? Does CD36 pharmacological inhibition affect preferentially the interaction with one specific ligand (i.e., FA vs oxLDL)? Given that most of the studies using blocking antibodies have been performed with an anti-CD36 IgA, is the antibody isotype relevant for the antitumoral effects? What are the adverse effects of chronic CD36 inhibition? Answering these questions will help to refine the search for better drug candidates for modulating CD36 function. Given the importance of CD36 in tumor progression, we foresee that this information will be available in the near future.
